# Vancomycin Heteroresistance Is Associated with Reduced Mortality in ST239 Methicillin-Resistant *Staphylococcus aureus* Blood Stream Infections

**DOI:** 10.1371/journal.pone.0021217

**Published:** 2011-06-21

**Authors:** Sebastiaan J. van Hal, Mark Jones, Iain B. Gosbell, David L. Paterson

**Affiliations:** 1 Department of Microbiology and Infectious Diseases, Sydney South West Pathology Service – Liverpool Hospital, Sydney, New South Wales, Australia; 2 Microbiology and Infectious Diseases Unit, School of Medicine, University of Western Sydney, Penrith South, New South Wales, Australia; 3 Centre for Healthcare Related Infection Surveillance and Prevention, Queensland Health and School of Population Health, The University of Queensland, Brisbane, Queensland, Australia; 4 University of Queensland Centre for Clinical Research (UQCCR), Brisbane, Queensland, Australia; University of Complutense, Spain

## Abstract

**Background:**

Despite hVISA infections being associated with vancomycin treatment failure, no previous study has been able to detect a mortality difference between heteroresistant vancomycin intermediate *Staphylococcus aureus* (hVISA) and vancomycin susceptible *Staphylococcus aureus* (VSSA) bloodstream infections (BSI).

**Methodology:**

Consecutive methicillin-resistant *S. aureus* (MRSA) BSI episodes between 1996 and 2008 were reviewed. Patient demographics, clinical presentation, treatment and overall mortality at 30 days were extracted from the medical records. All isolates underwent vancomycin minimum inhibitory concentration (VMIC) testing by broth microdilution and Etest. hVISA was confirmed by population analysis profiling using the area under the curve method (PAP-AUC).

**Principal Findings:**

401 evaluable MRSA BSI episodes were identified over the 12 years. Of these, 46 (11.5%) and 2 (0.5%) were confirmed as hVISA and VISA by PAP-AUC respectively. hVISA predominantly occurred in ST239-like MRSA isolates with high VMIC (2 mg/L). Compared to VSSA, hVISA was associated with chronic renal failure (p<0.001), device related infections (haemodialysis access) (p<0.001) and previous vancomycin usage (p = 0.004). On multivariate analysis, independent predictors of mortality included age, presence of multiple co-morbidities, principal diagnosis, transit to ICU and severity of illness while infection related surgery and hVISA phenotype were associated with increased survival.

**Conclusions/Significance:**

The presence of hVISA is dependent on the appropriate interplay between host and pathogen factors. hVISA in ST239 MRSA is an independent predictor of survival. Whether these findings would be replicated across all MRSA clones is unknown and warrants further study.

## Introduction

Methicillin-resistant *Staphylococcus aureus* (MRSA) accounts for approximately 24% of all *S. aureus* blood stream infections (BSI) in Australia. Not only do these infections lead to significant morbidity and increased health-care costs, they are associated with 30 day mortality rates of approximately 30% [1], [2].

Vancomycin has been regarded as the mainstay of treatment for these infections [3]. The first isolates with reduced or intermediate vancomycin (VISA) susceptibility emerged in Japan in 1997 [4]. Shortly thereafter, a heteroresistant vancomycin *Staphylococcus aureus* (hVISA) phenotype was detected [5]. hVISA isolates are characterised by the presence of a resistant subpopulation typically at a rate of 1 in 10^5^ organisms and represents the intermediary stage between fully vancomycin susceptible *S. aureus* (VSSA) and VISA isolates.

hVISA and VISA isolates have since been recognized globally [6], [7]. However, the exact prevalence of hVISA remains difficult to ascertain as testing methodologies are not standardised. Using the currently accepted gold standard, population analysis profiling – area under the curve method (PAP-AUC), hVISA accounts for between 8 and 29% of all MRSA BSI episodes [6], [8].

The clinical significance of hVISA infections remains unclear [7]. In several retrospective studies, hVISA BSI was associated with higher rates of vancomycin treatment failure, longer duration of bacteraemia and high inocula infections such as infective endocarditis [9], [10], [11]. *In vitro*, the presence of heteroresistance is linked to an isolate's vancomycin minimum inhibitory concentration (MIC); principally occurring in MRSA isolates with MIC≥2 mg/L by E-test [12]. The significance of this association is suggested by several recent studies that demonstrate poorer outcomes (mortality) in patients with high MIC (≥2 mg/L) MRSA blood stream infections [12], [13], [14].

Despite all these associations, no study to date has been able to demonstrate a mortality difference between VSSA and hVISA BSI [7]. This may suggest reduced virulence of heteroresistance as demonstrated by animal data and reduced *in vitro* host immune responses [15], [16], [17]. Indirectly supporting this hypothesis is the reduced ability of hVISA to cause infection compared to VSSA isolates [18] and the lower risk of shock with high MIC (2 mg/L) bacteraemia episodes [13].

The aim of the current study is to correlate vancomycin susceptibility determined by PAP-AUC, and vancomycin MIC to morbidity and mortality of MRSA BSI. (This work has been presented in part at the 50^th^ Interscience Conference on Antimicrobial Agents and Chemotherapy [ICAAC], Boston, MA, 12–15 September, 2010) [19]

## Results

During the 12 year period, 409 MRSA bacteraemia episodes were identified. Eight episodes (1.9%) were excluded due to the unavailability of the patients' medical records.

### Microbiological characteristics

353 (88%) VSSA; 46 (11.5%) hVISA and 2 (0.5%) VISA episodes were classified by PAP-AUC from the remaining 401 episodes. An association between increasing vancomycin MIC (irrespective of method used) and the presence of heteroresistance existed (Table 1) (p<0.001) with the majority of hVISA (82.6%; 38/46) episodes having a MIC 2 mg/L by broth microdilution. No evidence of MIC creep was detected over the study period (data not shown). PFGE was able to categorise 390 (97.3%; 390/401) episodes with 100% of hVISA isolates resembling ST239-MRSA-III clone (Table 1).

**Table 1:** Microbiological characteristics of *Staphylococcus aureus* blood stream infection episodes classified by population analysis profiling (PAP-AUC).

	%hVISA	Isolates no. (%)		
		hVISA (n = 46)	VSSA (n = 353)	p-value
Vancomycin MIC				
Broth microdilution				***<0.001***
≤0.5 mg/L	2.9	1 (2.2)	34 (9.6)	
1 mg/L	1.5	4 (8.7)	270 (76.5)	
1.5 mg/L	6.8	3 (6.5)	41 (11.6)	
2 mg/L	84.4	38 (82.6)	7(2)	
4 mg/L	0.0	0 (0)	1 (0.3)	
Etest				***<0.001***
≤0.5 mg/L	0	0 (0)	56 (15.9)	
0.75 mg/L	1.3	1 (2.2)	75 (21.2)	
1 mg/L	1.9	2 (4.3)	105 (29.7)	
1.5 mg/L	10.2	11 (23.9)	97 (27.5)	
2 mg/L	60.0	30 (65.2)	20 (5.7)	
3 mg/L	100	2 (4.3)	0 (0)	
PFGE type				
ST239-like*		46 (100)	265 (75.1)	***0.025***
ST 22-like		0	40 (11.3)	
ST 1-like		0	18 (5.1)	
ST 93-like		0	10 (2.8)	
ST 30-like		0	9 (2.5)	
Unclassified		0	11 (3.1)	

*Pulse field gel electrophoresis (PFGE) with *Sma*I restriction. Isolates were classified based on band characteristics compared to known multilocus sequence typed isolates.

The proportion of vancomycin susceptible and heteroresistant isolates by method of minimum inhibitory concentration determination and Pulse Field Gel Electrophoresis (PFGE) typing.

### Clinical characteristics

The two episodes of bacteraemia with VISA were secondary to a prosthetic joint infection and a post-surgical skin and soft tissue infection. Both patients were effectively treated with vancomycin, surgical debridement and several months of oral antibiotics. At 30 days, both patients were alive. Both VISA episodes were excluded from the remaining analysis. However, all of the subsequent associations detected between hVISA and VSSA BSI episodes remained significant if VISA episodes were included and considered as hVISA.

Clinical characteristics of hVISA compared to VSSA episodes are shown in Table 2. Patient demographics, mode of acquisition, location of the original blood culture and severity of illness (APACHE II score) did not differ between the two groups. hVISA episodes were significantly more likely to be associated with patients on dialysis for chronic renal failure (p<0.001) and be secondary to a haemodialysis access device (p<0.001 vs. p = 0.26 for all other principle diagnoses) compared to VSSA patients. Prior vancomycin therapy (in the preceding 30 days) was more likely to have occurred in hVISA episodes (p = 0.004). In contrast, there was no difference between the two groups with respect to antibiotic therapy chosen to treat the current episode, with vancomycin monotherapy used as the principal therapy in 83.7% (334/399) of patients. Similarly, vancomycin levels did not differ between the two groups with the median vancomycin level 12.7 and 14 mg/L for hVISA and VSSA episodes respectively (p = 0.086). No additional differences with respect to patient management were detected with infection related surgery (21.7% for hVISA vs. 17.8% for VSSA; p = 0.52) or device (line or haemodialysis access) removal similar in both groups (46.2 & 84.6% for hVISA vs. 57% & 88.6% for VSSA within 2 and 5 days from the positive blood culture; p = 0.31 & 0.57 respectively).

**Table 2:** Demographic and clinical features of *Staphylococcus Aureus* bacteraemia associated with heteroresistance compared to vancomycin susceptible episodes.

	hVISA(n = 46)	VSSA(n = 353)	P value
Patient demographics:			
Male sex	28 (60.9)	244 (69.1)	0.31
Median Age (IQR in years)	60 (50–70)	60 (50–70)	0.59
Co-morbidities:			
Chronic renal failure	20 (43.4)	62 (17.6)	***<0.001***
Congestive cardiac failure	14 (30.4)	81 (23.0)	0.27
Diabetes	15 (32.6)	97 (27.5)	0.49
Solid organ cancer	8 (17.4)	58 (16.4)	0.83
Haematological malignancy	4 (8.7)	50 (14.2)	0.37
Intravenous Drug Use	1 (2.2)	16 (4.5)	0.71
Recipient of Corticosteroids	7 (15.2)	60 (17.0)	1.0
Mean Charlson Weighted Index (SD)	2.3 (±1.5)	2.0 (±1.5)	0.12
Mode of acquisition:			0.18
Nosocomial	27 (58.7)	218 (61.8)	
Health Care Associated	18 (39.1)	104 (29.5)	
Community-acquired	1(2.2)	31 (8.8)	
Location of BSI:			0.94
In ICU	10 (21.7)	74 (20.9)	
Transit to ICU	4 (8.7)	38 (10.8)	
Ward	32 (69.6)	241 (68.3)	
Principal diagnosis:			***0.006*** 1
Infective endocarditis	2 (4.3)	13 (3.7)	
Skin and Soft Tissue infection	1 (2.2)	31 (8.8)	
Osteoarticular infection	3 (6.5)	34 (9.6)	
Post-surgical	8 (17.4)	40 (11.3)	
Pneumonia	2 (4.3)	50 (14.2)	
Device (line related)	9 (19.6)	66 (18.7)	
Device (haemodialysis access)	17 (37)	48 (13.6)	
Device (Other)	2 (4.3)	21 (5.9)	
No focus found	1 (2.2)	39 (11)	
Other	1 (2.2)	11 (3.1)	
Severity of illness:			
Median APACHE II score (IQR)	14 (10–16)	13 (9–18)	0.76
Laboratory parameters			
Median C- reactive protein (IQR in mg/L)	167 (101–232)	140 (59–242)	0.51
Median Albumin (IQR in g/L)	31 (20–34)	29 (24–34)	0.75
Antibiotic therapy			***0.093***
No treatment	2 (4.3)	20 (5.7)	
Alternative antibiotic therapy	1 (2.2)	42 (11.9)	
Vancomycin as principal therapy	43 (93.4)	291 (82.4)	
Vancomycin therapy			
Previous vancomycin use	15 (32.6)	54 (15.3)	***0.004***
Initial vancomycin level (IQR in mg/L)	9.8 (5.9–14)	10.2 (6.5–15.3)	0.40
Median vancomycin level (IQR mg/L)	12.7 (9.9–16)	14 (10.8–17.6)	***0.086***
Outcomes:			
Persistent bacteraemia	5/18 (27.8)*	40/134 (29.8)*	1.0
Metastatic complications	3 (6.5)	12 (3.4)	0.40
Infection related surgery	10 (21.7)	63 (17.8)	0.52
Median hospital LOS (IQR in days)	45 (19–107)	31 (15–54)	***0.033***
Overall 30 day mortality	5 (10.9)	110 (31.2)	***0.003***

2 VISA episodes excluded.

NOTE: Data are no. (%) of episodes, unless otherwise indicated.

1 There was a significant difference between the two groups with respect to the principal diagnosis distribution. This difference was due to haemodialysis access device (p<0.001) with all other diagnoses similar between the 2 groups (p = 0.23).

*Only 18 hVISA and 134 VSSA bacteraemia episodes had repeat blood cultures performed, 5 days after the initial isolate.

### Outcomes

In the 152 episodes (38.1%; 152/399) that had additional blood cultures performed; hVISA episodes were not more likely to be persistently bacteraemic (5 of 18; 27.8%) compared to VSSA episodes (40 of 134; 29.8%). Compared to VSSA, hVISA episodes had similar rates of metastatic complications but were associated with longer hospital admissions (an additional 14 days; p = 0.033).

**Figure 1:**
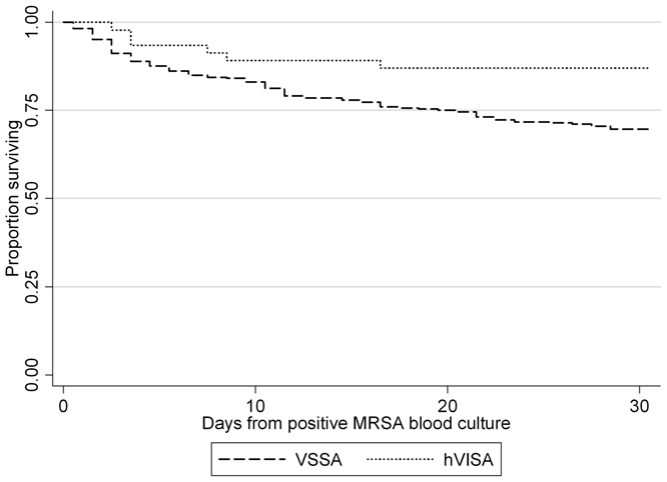
Kaplan Meyer survival curves 30 days from the initial positive blood culture bottle for Methicillin-resistant *Staphylococcus aureus*. Patients with vancomycin heteroresistance (hVISA) were significantly less likely to die compared to patients with vancomycin susceptible (VSSA) blood stream episodes (OR of 0.27; 95% CI 0.09–0.83; p = 0.022).

Overall mortality at 30 days was 29% (115/401) with hVISA episodes associated with a significantly lower rate of mortality at 30 days (10.9%) compared to VSSA episodes (31.2%; p = 0.003) (Table 2 and Figure 1). Multiple factors including age, the presence of congestive cardiac failure, solid organ cancer, transit to ICU, principle diagnosis, severity of illness (APACHE II score), lack of infection related surgery and VSSA were all predictors of mortality at 30 days (Table 3) on univariate analysis. Vancomycin MIC by E-test or broth microdilution (data not shown) did not predict mortality in the overall or the vancomycin treated group. Clonal type by PFGE did not predict outcome (p = 0.78).

**Table 3:** Univariate analysis of clinical features associated with overall 30 day mortality in *Staphylococcus aureus* blood stream infection episodes.

Characteristic	Survived (n = 284)	Died (n = 115)	P-value
Patient demographics:			
Male sex	197 (69.4)	75 (65.2)	0.48
Median Age (IQR in years)	61 (46–72)	72 (64–78)	***<0.001***
Co-morbidities:			
Chronic renal failure	64 (22.5)	18 (15.7)	0.13
Congestive cardiac failure	55 (19.4)	40 (34.8)	***0.001***
Diabetes	83 (29.2)	29 (25.2)	0.46
Solid organ cancer	48 (16.9)	28 (24.3)	***0.011***
Haematological malignancy	33 (11.6)	21 (18.3)	0.10
Intravenous Drug Use	16 (5.6)	1 (0.9)	***0.030***
Recipient of Corticosteroids	45 (15.8)	22 (19.1)	0.46
Mean Charlson Weighted Index (SD)	1.9 (±1.5)	2.4 (±1.6)	***0.004***
Mode of acquisition:			***0.029***
Nosocomial	169 (59.5)	76 (66.1)	
Health Care Associated	86 (30.3)	36 (31.3)	
Community-acquired	29 (10.2)	3 (2.6)	
Location of BSI:			***0.004***
In ICU	55 (19.4)	29 (25.2)	
Transit to ICU	22 (7.7)	20 (17.4)	
Ward	207 (72.9)	66 (57.4)	
Principal diagnosis:			***<0.001*** 1
Infective endocarditis	6 (2.1)	9 (7.8)	
Skin and Soft Tissue infection	27 (9.5)	5 (4.3)	
Osteoarticular infection	32 (11.3)	5 (4.3)	
Post-surgical	38 (13.4)	10 (8.7)	
Pneumonia	20 (7)	32 (27.8)	
Device (line related)	62 (21.8)	13 (11.3)	
Device (haemodialysis access)	53 (18.7)	12 (10.4)	
Device(other)	20 (7)	3 (2.6)	
No focus found	21 (7.4)	19 (16.5)	
Other	5 (1.8)	7 (6.1)	
Severity of illness:			
Median APACHE II score (IQR)	12.5 (8–16)	19.1 (13–25)	***<0.001***
Laboratory parameters:^2^			
Median Albumin (IQR in g/L)	29.4 (24–34)	27.3 (22–33)	***0.017***
Antibiotic Therapy:			
No treatment	6 (2.1)	39 (33.9)	NA
Alternative antibiotic therapy	18 (6.3)	2 (1.7)	
Vancomycin as principal therapy	260 (91.5)	74 (64.3)	
Vancomycin treated group:^3^			0.63
VMIC 1	149 (52.5)	45 (39.1)	
VMIC 1.5	72 (25.4)	21 (18.2)	
VMIC 2	39 (13.7)	8 (7)	
Phenotype:^4^			***0.003***
hVISA by PAP	41 (14.4)	5 (4.3)	
VSSA by PAP	243 (85.6)	110 (95.7)	
Outcomes:^2^			
Infection related surgery	64 (22.5)	7 (6.1)	***<0.001***
Metastatic complications	13 (4.6)	2 (1.7)	0.25

2 VISA episodes excluded.

NOTE: Data are no. (%) of patients, unless otherwise indicated.

NA: To prevent survivor treatment bias, analysis of no treatment was not performed.

1 The principal diagnosis distribution in deceased patients differed significantly compared to patients that survived. This difference was due to a diagnosis of infective endocarditis, pneumonia, device (other) related infection and no identifiable focus. The strength of each of these factors can be found in Table 4.

2 C-reactive protein and persistent bacteraemia variables were not included in mortality analyses due to a high proportion of missing data.

3 The effect of vancomycin MIC determined by E-test on mortality for patients treated with vancomycin. Similar results were obtained for the total group and when MIC was determined by broth microdilution.

4 Heteroresistant vancomycin intermediate (hVISA) and vancomycin susceptible (VSSA) Staphylococcus aureus phenotypes determined using population analysis profiling area under the curve method.

Independent predictors of mortality in logistic regression analysis included age (Odds ratio [OR] 1.03 per year; 95% confidence interval [CI] 1.01–1.05; p = 0.005), the presence of multiple co-morbidities (Charlson weight index; OR 1.24; 95% CI 1.03–1.5; p = 0.023), transit to ICU (OR 2.82; 95% CI 1.25–6.35; p = 0.012), principle diagnosis (i.e. infective endocarditis [OR 6.2; 95 CI 1.79–21.5], pneumonia [OR 5.23; 95% CI 2.5–10.9] and the absence of a identified focus [OR 3.88 95% CI 1.7–8.89]; p<0.001) and severity of illness (APACHE II score; OR 1.11 per point; 95% CI 1.07–1.15; p<0.001). (Table 4). In contrast, both hVISA phenotype (OR 0.27; 95% 0.09–0.83; p = 0.022) and infection related surgery (OR 0.27; 95% 0.10–0.74; p = 0.011) were strong predictors of survival while vancomycin MIC did not predict outcome. The final multivariate model was able to distinguish between survivors and non-survivors at 30 days with reasonable accuracy (c-statistic of 0.85) with no evidence of a lack-of-fit (p = 0.91) determined by the Hosmer-Lemeshow test.

**Table 4:** Multivariate analysis of risk factors associated with overall 30 day mortality in *Staphylococcus Aureus* blood stream infection episodes.

Risk Factor	p-Value	Odds Ratio	95% CI
Age, per year	***0.005***	1.03	1.01–1.05
Co-morbidities:			
Chronic renal failure	0.24		
Congestive cardiac failure	0.80		
Solid organ cancer	0.23		
Haematological malignancy	0.45		
Intravenous Drug Use	0.47		
Charlson Weighted Index	***0.023***	1.24	1.03–1.50
Mode of acquisition:	0.18		
Location of BSI:			
Transit to ICU	***0.012***	2.82	1.25–6.35
Principal diagnosis:	***<0.001***		
Reference^1^		1.00	
Infective endocarditis		6.20	1.79–21.5
Pneumonia		5.23	2.50–10.9
No focus found		3.88	1.70–8.89
Device (other)		3.34	1.15–9.67
Severity of illness:			
APACHE II score, per point	***<0.001***	1.11	1.07–1.15
Laboratory parameters			
Median Albumin (in g/L)	0.67		
Vancomycin treated group^2^	0.67		
VMIC 1			
V MIC 1.5			
V MIC 2			
Phenotype^3^			
hVISA by PAP-AUC	***0.022***	0.27	0.09–0.83
Outcomes:			
Infection related surgery	***0.011***	0.27	0.10–0.74

1 Reference group composed of all principle diagnoses with similar 30 day mortality.

2 The effect of vancomycin MIC determined by E-test on mortality for patients treated with vancomycin. Similar results were obtained for the total group and when MIC was determined by broth microdilution.

3 Heteroresistant vancomycin intermediate (hVISA) *Staphylococcus aureus* phenotype determined using population analysis profiling area under the curve method.

Vancomycin MIC was further assessed in vancomycin monotherapy treated VSSA episodes only (291 of 353). High vancomycin MIC (of 2 mg/L by broth microdilution) isolates had a non-significant lower mortality (22%; 2/9) compared to episodes with a lower MIC<2 mg/L (31%; 108/344) (p = 0.73).

## Discussion

This is one of the largest cohorts of MRSA BSI evaluating outcomes where all isolates have undergone the gold standard test (PAP-AUC) for the presence of heteroresistance. Although rates of hVISA vary widely by geographical region, testing methodology and study population selected [7]; our rate of 12%, is similar to two other Australian studies examining consecutive BSI isolates [9], [18]. Prior vancomycin usage was an important risk factor for the development of heteroresistance while drug exposure; determined by vancomycin serum trough levels, was not. This is not surprising as vancomycin trough is a poor surrogate for total drug exposure [20].

Morbidity was considerable in patients with hVISA infection with an average increase of hospital stay of 14 days compared to VSSA episodes. This is despite similar rates of treatment failure, defined as bacteremic persistence, between the two groups. This finding supports assertions that hVISA may reflect the consequence rather than the cause of treatment failure [7], [21], [22]. This has implications with respect to selecting isolates for further testing; as PAP-AUC is labour intensive and expensive [21], [22]. A recent review suggested a clinically based algorithm prompted by persistent bacteraemia [21]. Based on our data, the need to confirm hVISA phenotype may not be necessary as clinical management decisions could be determined by the principal diagnosis and documented treatment failure or bacteraemia persistence [6], [10], [21].

Overall 30 day mortality was significantly lower for hVISA infections (11% vs 31% for VSSA). This may reflect host factors as hVISA infections were significantly more likely to occur in dialysis dependent chronic renal failure patients. In addition, device related infections predominated in hVISA episodes, a principal diagnosis which generally is associated with better outcomes especially if the access devices are removed [2].

Independent predictors of mortality included age, presence of multiple co-morbidities, severity of illness, transit to ICU and principal diagnosis. All these factors have been previously found to be associated with increased mortality with transit to ICU a surrogate marker of severe sepsis [2], [13]. However, unlike previous studies, infection-related surgery was found to be protective. This variable is strongly confounded by a patients' ability to undergo a procedure with the sickest patients excluded from surgery. However, an alternative explanation is that patients who have extensive debridement and source control are more likely to survive due to the increased effectiveness of treatment and antibiotics [23]. As our data was collected retrospectively, we were unable to distinguish between these two possibilities. Nevertheless, given the poor outcomes with MRSA BSI this intervention should be explored further.

Similarly, contrary to previous studies [9], [10], [12], the presence of vancomycin heteroresistance was independently associated with a decreased 30 day mortality compared to VSSA BSI episodes. The most likely explanations for these findings are the interplay between host and pathogen specific factors. MRSA requires an enduring niche with sustained pressure for hVISA emergence. However, the propensity for susceptible bacterial populations to transform to a hVISA phenotype various greatly between MRSA clones. Clonal complex 8 (CC8), of which ST239 is a member, is documented to transform and maintain the resistance phenotype easily and thus is overrepresented (78% of hVISA isolates) in recent study of MRSA infective endocarditis [6]. These factors would explain the strong association of hVISA in our chronic renal failure patients; secondary to the convergence in dialysis units, of high vancomycin exposed patients and a high MRSA burden. Moreover, this clarifies the reason for hVISA being limited to the predominant MRSA hospital clone in our setting, ST239. Whether these findings would be replicated in similar populations groups, with other MRSA clones, is unknown and warrants further study.

Alternatively, our observations support the reduced pathogenicity that may occur with hVISA infections [16], [17] and corroborates the significance of the *in vitro* genomic alterations in the major virulence regulator (*agr*) of vancomycin heteroresistant *S.aureus* isolates [24], [25]. Additionally, the specificity of hVISA testing methods vary, Macromethod Etest the most common method used in BSI studies [12], [13] is likely to overestimated the burden of heteroresistance [22] and thus mask the real effect of this phenotype.

Given that hVISA episodes occurred predominantly in isolates with high vancomycin MICs, no association between outcome and vancomycin MIC was detected. At first glance, this is likewise contrary to previous studies showing poorer outcomes with high but susceptible vancomycin MIC bacteremic episodes [12], [13], [26]. However, our data probably reflects specific ST239 MRSA clonal issues. Alternatively, as hVISA prevalence varies between geographical locations [7] with lower rates in non-Oceanic institutions [6]; MIC data may pertain to VSSA infections only [12]. Additionally, pathogen factors may play an important role in determining outcomes in these patients as vancomycin MIC has been found to be an independent predictor of mortality in flucloxacillin treated methicillin-sensitive *S. aureus* episodes. [27].

Irrespective of the differences, our data shows that hVISA or MIC associations with outcomes are far more complex than first assumed. There are probably unidentified pathogen specific factors that direct these responses and that these associations do not prove causality. Thus it would be prudent to study these issues further before relegating vancomycin to second line therapy.

This study has several limitations. Data was gathered retrospectively. However, as consecutive BSI isolates were examined over an extended period with a small exclusion rate (2.9%), we were able to minimise this bias. Furthermore, as clinicians were unaware of the hVISA status, therapy was not altered based on presence of heteroresistance and thus adds weight to our findings. Clinical management could have altered over time with more aggressive vancomycin dosing, especially in the latter part of the study leading to a difference. However, no trends, MIC creep or significant median trough vancomycin levels changes were detected in our study over time (data not shown). Although the hVISA sample size is relatively small, our study is the largest comparative study of hVISA BSI episodes and importantly, uses the currently accepted gold standard, PAP-AUC, for detection [21].

Finally, vancomycin tolerance (determined by minimum bacteriocidal to MIC ratios) was not determined in our study. As this variable is known to affect vancomycin treatment responses [28], [29] it may have impacted our results. This would be especially true for VSSA responses as up to 15% of these isolates can be associated with tolerance and thus suboptimal responses [30]. Conversely hVISA is not exclusively associated with tolerance [30] and thus this variable warrants further research.

In summary, this study makes several key observations. The presence of heteroresistance is dependent on the appropriate interplay between host and pathogen. Factors such as age, principal diagnosis, presence of co-morbidities, transit to ICU and severity of illness were all strong predictors of mortality. In contrast, infection-related surgery and ST239 MRSA hVISA phenotype were independent predictors of survival. Further studies are required to validate these findings and tease out the complexities associated with pathogen specific factors.

## Materials and Methods

### Setting

Liverpool Hospital, Sydney, Australia is a 600-bed teaching hospital that provides medical and surgical care to the surrounding community of approximately 850 000 people.

### Microbiological methods

During the 12 year study period, all positive blood cultures (BacT/ALERT 3D®; bio-Mérieux, Australia) with MRSA, identified according to conventional techniques were stored (−80°C). 458 isolates were identified of which 409 represented the initial isolate for each episode. Further isolate testing, following growth on horse blood agar, occurred under blinded conditions without knowledge of the clinical outcomes. Vancomycin MIC was determined by E-test using a 0.5 McFarland inoculum (AB BIODISK, Solna, Sweden) evenly streaked onto Mueller-Hinton Agar (BBL, Becton Dickinson, MD). Broth microdilution was performed in accordance with The Clinical Laboratory Standards Institute method using the current susceptible breakpoint of ≤2 mg/L and 4–8 mg/L to define VSSA and VISA isolates respectively [31]. In addition, all isolates were assessed by PAP-AUC as previously described [32]. In brief, a bacterial density of 0.5 McFarland was inoculated onto brain heart infusion agar (BHIA) plates containing increasing doses of vancomycin (0 to 6 ug/ml). Colony counts at 48 hours were plotted against vancomycin concentration with the AUC calculated. An isolate was defined as VSSA, hVISA or VISA if the ratio of the obtained AUC to the reference strain (Mu3; ATCC 700698) was <0.9; 0.9–1.3; and >1.3 respectively.

### Pulse-field gel electrophoresis

Pulse-field gel electrophoresis (PFGE) with *Sma*I restriction was performed on all isolates and analysed as previously described [33]. Isolates were classified based on band characteristics compared to known multilocus sequence typed isolates (supplied by G. Coombs, Royal Perth Hospital, Perth, Western Australia).

### Patient selection

All consecutive episodes of MRSA bacteraemia diagnosed in adult patients (>15 years of age) between 1/1/1996 and the 31/12/2008 were eligible for inclusion in the study. Standard information was retrospectively obtained from the laboratory information system and the patients' clinical records. Patient demographics (age, sex and admission date), the presence of co-morbidities, place of onset and location of infection, principle diagnosis, need for intensive care and treatments were extracted. The severity of illness was determined by the APACHE II score within 48 hrs of the initial obtained blood culture bottle which subsequently isolated MRSA. Only antibiotics to which the isolate was sensitive were considered with the principal antibiotic prescribed for each episode obtained from the medication charts. Vancomycin usage in the preceding 30 days was similarly obtained. Glycopeptide drug levels taken during treatment were retrieved from the laboratory information system and used to calculate the initial and median vancomycin levels.

### Definitions

A single MRSA BSI episode was defined as one or more positive blood cultures with systemic manifestations of infection, such as fever, chills with or without local signs and symptoms. Bacteraemia episodes were considered to be persistent if the repeated blood cultures remained positive following 5 days of appropriate antibiotics (e.g. vancomycin). Co-morbidity was defined as a pre-existing medical condition present on admission that could predispose a patient to an infection, for example diabetes, renal failure requiring dialysis or administration of immunosuppressive agents within the preceding 30 days. These were scored using the Charlson weighted index [34]. Bloodstream infections were considered hospital acquired (HA) when the positive blood cultures were obtained >48 hours after admission. The remaining episodes were classified as either community acquired (CA) or healthcare associated (HCA) based on contact with the healthcare system within the previous 30 days. The principal diagnosis was determined using the Centre for Disease Control definitions [35]. Mortality was defined as death that occurred within 30 days of the onset of bacteraemia. Attributable mortality was not determined to avoid introducing any interpretative bias.

### Statistical analysis

SAS version 9.2 for Windows (SAS Institute, Cary, NC) and Stata version 10.1 for Windows (StataCorp LP, College Station, TX) were used for statistical analysis. Fisher's Exact Test and Wilcoxon 2-sample test was used for categorical and continuous data when appropriate. All-cause mortality at 30 days post-admission was modelled using logistic regression and survival analysis. To determine independent predictors of mortality, vancomycin MIC and variables with a univariate p value<0.2 were considered a priori to be included in the multivariate model. A stepwise approach with backwards elimination was then used to remove statistically non-significant variables until the final “best” model was obtained. CRP and persistent bacteraemia variables were not included in mortality analyses due to a high proportion of missing data. Goodness-of-fit of the final logistic models were assessed using Hosmer-Lemeshow test and c-statistic which indicates how well the model distinguishes between survivors and non-survivors where 0.5 indicates a model that is not predictive and 1.0 indicates a model that predicts perfectly. Only the clinical and microbiological characteristics associated with the initial blood stream isolate for each episode were used in the analysis.
